# Genomic and proteomic profiling of *GATA3* mutant metastatic hormone receptor-positive breast cancer and impact on clinical outcomes

**DOI:** 10.1007/s10549-025-07710-w

**Published:** 2025-05-29

**Authors:** Arielle J. Medford, Marko Velimirovic, Yifat Gefen, Andrzej Niemierko, Lorenzo Gerratana, Andrew A. Davis, Katherine Clifton, Jennifer Keenan, Emily Podany, Whitney L. Hensing, Carolina Reduzzi, Charles S. Dai, Lesli A. Kiedrowski, Laura M. Spring, Leif W. Ellisen, Robert C. Doebele, Massimo Cristofanilli, Gad Getz, Aditya Bardia

**Affiliations:** 1 Massachusetts General Hospital & Harvard Medical School, Boston, MA USA; 2 Cancer Program, Broad Institute of Massachusetts Institute of Technology and Harvard, Cambridge, MA USA; 3 Department of Hematology/Oncology, Cleveland Clinic, Cleveland, OH USA; 4 Department of Medical Oncology, CRO Aviano, National Cancer Institute, IRCCS, Aviano, Italy; 5 Division of Oncology, Department of Medicine, Washington University School of Medicine, St. Louis, MO USA; 6 Weill Cornell Medicine, 420 E 70 th St, LH 204, New York, NY 10021 USA; 7 Department of Medical Oncology, Dana-Farber Cancer Institute, Harvard Medical School, Boston, MA USA; 8 Department of Medical Affairs, Guardant Health, Redwood City, CA USA; 9 Rain Therapeutics, Newark, CA USA; 10 David Geffen School of Medicine at UCLA & UCLA Health Jonsson Comprehensive Cancer Center, Los Angeles, CA 90024 USA

**Keywords:** Circulating tumor DNA, Liquid biopsy, Genomics, Breast cancer

## Abstract

**Purpose:**

*GATA3* mutations are among the most common alterations in hormone receptor-positive (HR+) breast cancer (BC), yet these have no targeted therapies. MDM2 is an E3 ubiquitin ligase that targets p53 for degradation, and pre-clinical data suggests MDM2 inhibition may effectively treat *GATA3*^mut^ HR+ BC. The *GATA3* co-mutational landscape has been described only in primary BC tissue, and the mechanism of MDM2-driven efficacy is incompletely understood.

**Experimental design:**

Circulating tumor DNA (ctDNA) was assessed for *GATA3* mutations via targeted sequencing. Associations with co-alterations and clinical/pathologic factors were estimated using Pearson's chi-squared test, two-sample Wilcoxon rank-sum, and multivariable logistic regression. Impact on survival was analyzed using multivariable Cox regression analysis. Tissue-based data from the Clinical Proteomic Tumor Analysis Consortium (CPTAC) database was evaluated for expression and phosphorylation of GATA3 and associated proteins.

**Results:**

Among 609 patients with HR + /HER2− MBC, ctDNA detected non-synonymous *GATA3* variants ctDNA in 69 (11%) patients, and the genomic landscape was unique from tissue-based primary BC data; *GATA3*^mut^ were not mutually exclusive from *TP53*^mut^ (*p* = 0.30) or *PIK3CA*^mut^ (*p* = 0.52) and were associated with poorer survival on endocrine monotherapy. CPTAC analysis showed no difference in *GATA3* or breast cancer-associated gene abundance, however there was increased USP48 (LogFC = 0.76, FDR = 1.7 × 10^–5^), which stabilizes MDM2.

**Conclusion:**

The distinct landscape in *GATA3*^mut^ MBC ctDNA highlights critical information when assessing candidacy for targeted therapies. To our knowledge, this is the first ctDNA-based *GATA3*^mut^ landscape analysis in MBC. Furthermore, tissue-based proteomic analysis suggests mechanisms for endocrine resistance and sensitivity to MDM2 inhibition in HR+ /HER2− *GATA3*^mut^ BC.

**Supplementary Information:**

The online version contains supplementary material available at 10.1007/s10549-025-07710-w.

## Introduction

Metastatic breast cancer (MBC) remains one of the leading causes of cancer-related death, despite significant therapeutic advances [1]. Hormone receptor-positive (HR +) breast cancer is the most common subtype, and while endocrine therapy remains the backbone of HR + MBC treatment, an increasing number of targeted therapies are becoming options for patients with specific genomic variants (e.g., *PIK3CA*^mut^, *ESR1*^mut^, *AKT*^mut^, *PTEN*^mut^) [2–4]*.*

*GATA3* mutations are frequent in breast cancer, with an estimated prevalence of 10–18% in HR + MBC; the majority are loss-of-function mutations and are associated with poor response to endocrine therapy (ET) and poor survival, as described in our previous work [5]. Despite being one of the most prevalently altered genes in breast cancer, and being associated with poorer outcomes, to our knowledge there have been no attempts to therapeutically target *GATA3*^mut^ MBC [6–21]. The GATA3 protein is the most highly expressed transcription factor in breast luminal epithelial cells and is critical for mammary epithelial development and luminal identity [6–16, 22]. Functionally, it interacts with the estrogen receptor (ER) and FOXA1 to increase the transcription of genes in the ER axis, and its normal expression is associated with well-differentiated tumors with low metastatic potential. High GATA3 expression is also associated with favorable clinical prognostic features, including lymph node negativity, lower tumor grade, older age at diagnosis, and negative human epidermal growth factor receptor 2 (HER2) [23, 24]. Meanwhile, low GATA3 expression and protein dysfunction are associated with undifferentiated tumors, increased risk of metastasis, and poorer overall outcomes [12, 19, 23–30].

While GATA3 has not been pursued as a direct therapeutic target, data suggest its potential indirect druggability in *GATA3*^mut^ HR + breast cancer via inhibition of MDM2 (mouse double minute 2), an E3 ubiquitin-protein ligase that targets p53 for degradation [31]. Given the fact that MDM2 inhibition requires functional p53 (i.e., wildtype *TP53*), along with the fact that multiple other targeted therapies are approved or under study in the HR +/HER2− population, deeper knowledge of the genomic landscape of *GATA3*^mut^ disease will sharpen our understanding of the patient population that may benefit from targeted therapy informed by *GATA3* mutational status.

Prior analyses of the *GATA3* mutational landscape have been performed on tissue sequencing data from primary breast cancers, where patients have not been exposed to therapeutic pressures [18, 25, 32–38]. Liquid biopsy analysis via circulating tumor DNA (ctDNA) is a less invasive diagnostic procedure and is now in regular use in advanced breast cancer as part of the standard of care and as a tool to assess eligibility for genomically targeted clinical trials [39–41]. We aim to expand on our prior work and analyze the *GATA3*^mut^ mutational landscape based on ctDNA next generation sequencing (NGS), and furthermore, we analyze *GATA3*^mut^ proteomic data to understand the mechanism behind the efficacy of MDM2 inhibition in *GATA3*^mut^ HR +/HER2− breast cancer.

In this multi-institutional ctDNA-based study, we characterized the mutational landscape of *GATA3*^mut^ vs. *GATA3*^WT^ HR +/HER2− MBC, investigated the differences in patients’ clinicopathologic features, observed survival outcomes, and also used tissue-based data from the Clinical Proteomic Tumor Analysis Consortium (CPTAC) describe the proteomic landscape of *GATA3*^mut^ MBC. To our knowledge, our study is the first ctDNA-based analysis of the *GATA3* mutational landscape in advanced breast cancer.

## Materials and methods

### Patient selection and genomic analysis

Patients at the Massachusetts General Hospital and at Washington University in St Louis consented to have their ctDNA data collected and medical information used for research purposes via an IRB-approved protocol. ctDNA was analyzed via Guardant360 (Guardant Health, Inc; Redwood City, CA, USA), a next generation sequencing assay that analyzed up to 74 genes during the study period. Guardant360 is a CLIA-certified, CAP-accredited, NYSDOH-approved clinical assay that detects somatic single nucleotide variants (SNVs) in 73–74 genes (panel composition changed during the course of the study), and indels, somatic copy-number gains, and fusions in a subset of these genes as previously described [42]. At allele fractions > 0.25% for mutations and fusions, > 0.20% for indels, and copy numbers > 2.24 copies, the analytical sensitivity of this assay is ≧95%, and specificity is > 99.99%. The association of *GATA3*^*mut*^ and co-mutations as well as number of prior therapies was estimated using Pearson's chi-squared test for categorical variables, two-sample Wilcoxon rank-sum test for continuous variables, and multivariable logistic regression. Statistical analysis was conducted using STATA, version 16—Stata (RRID:SCR_012763), StataCorp LLC—Stata (RRID:SCR_012763). The OncoKB tool was used to annotate *GATA3* variants for oncogenicity [43].

### Proteomic analysis

For this study, we included tissue-based data from 73 out of the 121 available CPTAC BRCA HER2-negative samples; out of this cohort, 9 samples had *GATA3* mutations. We used the provided processed tables from the original study [44], specifically the median-MAD normalized log2-transformed protein and phosphosite expression tables. Differential expression was performed between mutated and unmutated samples and false discovery rate (FDR) was computed using the Benjamini–Hochberg procedure.

### Survival analysis

The impact of *GATA3*^*mut*^ and *GATA3*^*WT*^ detected via ctDNA on progression-free survival (PFS) and overall survival (OS) was analyzed using multivariable Cox regression analysis adjusting for age, number of prior therapies, visceral metastases, and de novo metastases, to account for variability in other clinical factors in this retrospective real-world cohort. PFS and OS were evaluated in the overall study population, as well as in subgroups of patients that received endocrine monotherapy (GATA3^mut^ *n* = 6; GATA3^WT^ *n* = 74), ET + CDK4/6 inhibitor (GATA3^mut^ *n* = 28; GATA3^WT^ *n* = 191), and chemotherapy (GATA3^mut^ n = 18; GATA3^WT^ *n* = 146). Radiographic progression was determined by RECIST criteria for patients who were participants in clinical trials. If patients were receiving standard of care therapy, progression was radiographically assessed by radiologists who were not aware of the results of ctDNA sequencing or aims of this study.

All outcomes analyses were performed using STATA, version 16—Stata (RRID:SCR_012763), StataCorp LLC—Stata (RRID:SCR_012763).

## Results

### Detection of *GATA3* mutations by plasma-based genotyping in HR + MBC

Among the 647 patients with HR + MBC in the ctDNA study cohort, 609 had HR +/HER2− MBC. Among the latter, there were 69 patients (11%) in whom ctDNA analysis identified a non-synonymous *GATA3* mutation. *GATA3* mutations were observed in a statistically significant larger percentage of patients with documented visceral disease (76.8% vs. 62.0%; *p* = 0.016) and de novo metastatic disease (33.3% vs. 20.7%; *p* = 0.018) compared to *GATA3*^*WT*^; no other distinguishing clinical characteristics, including age at metastatic diagnosis, number of prior therapies, and progesterone receptor status, were observed (see Table 1 for clinical characteristics & mutational status of the cohort). Of note, when comparing only patients with frameshift or nonsense *GATA3* mutations (annotated as likely oncogenic or known oncogenic via the OncoKB tool, *n* = 55), observations and significance were similar to the above (Supplemental Table 1). A separate analysis showed no correlation with HER2 status and *GATA3* mutations, but given the more aggressive phenotype and different treatment approach in HER2 + MBC, this subtype was otherwise excluded from this analysis. Among these 69 patients with HR +/HER2− *GATA3*^mut^ MBC, 49 (71%) patients identified as White and 14 (20%) as Black. Patients had received a median of 2 prior lines of therapy in the metastatic setting (range 0–9).

**Table 1:** Patient characteristics in patients with *GATA3*^WT^ and *GATA3*^mut^ HR +/HER2− MBC

Patient characteristics	*GATA3* wildtype (*n* = 540)	*GATA3* mutation (*n* = 69)	*p*-value
Age, median (IQR)	61.3 (52.5, 69.7)	59.1 (48.6, 65.4)	0.060
De novo metastases	112 (20.7)	23 (33.3%)	0.018
Visceral metastases	335 (62.0%)	53 (76.8%)	0.016
Number of prior therapies, median (IQR)	2 (1, 4)	2 (1, 5)	0.055
*TP53* co-mutation	215 (39.8%)	23 (33.3%)	0.30
*PIK3CA* co-mutation	213 (39.4%)	30 (43.5%)	0.52

Next, we aimed to characterize *GATA3* mutations and their location within the gene. The GATA3 protein is encoded by 444 amino acids, which encompass 6 exons, 5 of which are coding; the gene also contains two transactivating domains and two Zinc finger (ZnFn) motifs. [13] ZnFn 1 stabilizes DNA binding, while ZnFn 2 binds DNA at the *GATA3* motif. [45] Prior tissue-based analyses have found most *GATA3* mutations to occur at the splice sites between exons 4/5 and 5/6, as well as within exon 5 (including Zn finger 2) and exon 6, the majority of which were frameshift mutations and annotated as likely oncogenic per the OncoKB tool [14, 25, 32, 33, 43, 46].

**Fig. 1:**
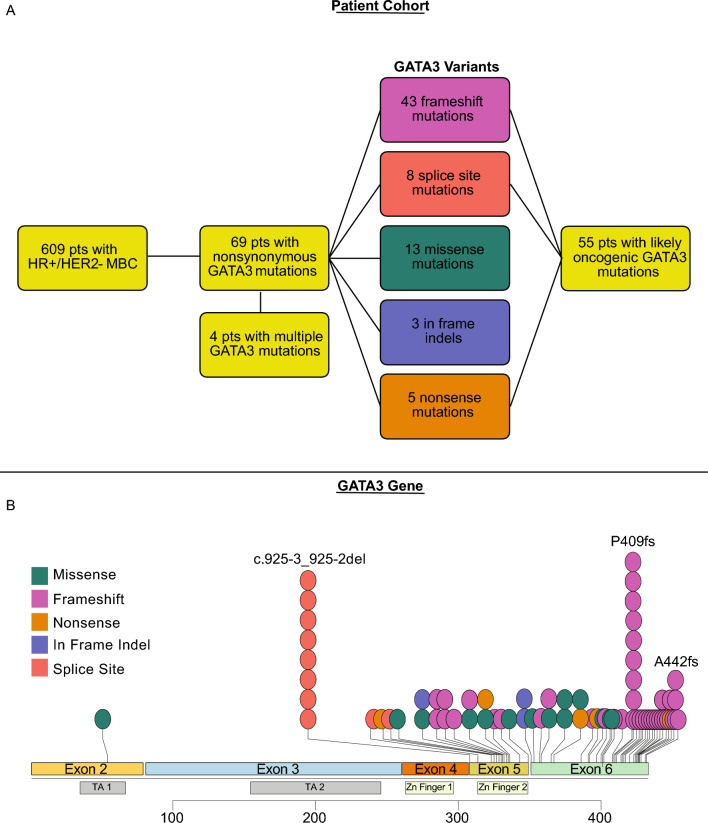
*GATA3* gene mutation overview—**A** Flowchart of patient ctDNA cohort harboring *GATA3* gene mutations; **B** Mutational distribution within the *GATA3* gene

In our ctDNA analysis, among these 69 *GATA*.^mut^ patients we found 73 *GATA3* mutations: 18 (26%) occurred in exon 5, all but three of which were in the second zinc finger, 8 (11%) were c.925-3_925-2 del splice site variants, and 46 (67%) were in exon 6. In terms of mutation type, 43 (62%) were frameshift mutations, 14 (20%) were missense mutations, and 5 (7%) were nonsense mutations (Fig. 1B). Comparison to breast cancer variants in the Catalog Of Somatic Mutations In Cancer (COSMIC) database, a tissue-based cancer mutation repository, also showed the majority of variants being frameshift mutations (Supplemental Fig. 1). Median variant allele fraction (VAF) of the *GATA3* mutations was 1.0% (IQR 0.34–5.5%). To approximate clonality, we calculated the fraction of the alteration of interest relative to the alteration in the ctDNA sample with the highest VAF; we used 30% as a cutoff. Four patients had two *GATA3* mutations with comparable VAFs (listed in parentheses), consistent with them being two clonal hits (based on above criteria to estimate clonality as ratio of VAF over highest VAF in the sample): F392L (6.40%)/P409fs (6.20%), 336* (1.29%)/435* (0.71%), W329R (0.8%)/A395 V (0.1%), and A442fs (2.9%)/R353 T (2.4%). While the Guardant360 test is not intended to report germline mutations, we set a VAF cutoff of 40% to identify potential germline *GATA3* alterations, which result in HDR syndrome (hypoparathyroidism, deafness, renal dysfunction) [47, 48]. By the above criterion, no *GATA3* alterations appeared to be germline (i.e., all VAFs were under 40%). Fifty-five patients (80%) were determined to have likely oncogenic variants via OncoKB, which in this dataset included all frameshift, nonsense, and splice site variants (Fig. 1B) [43].

### *GATA3* mutational landscape

**Fig. 2:**
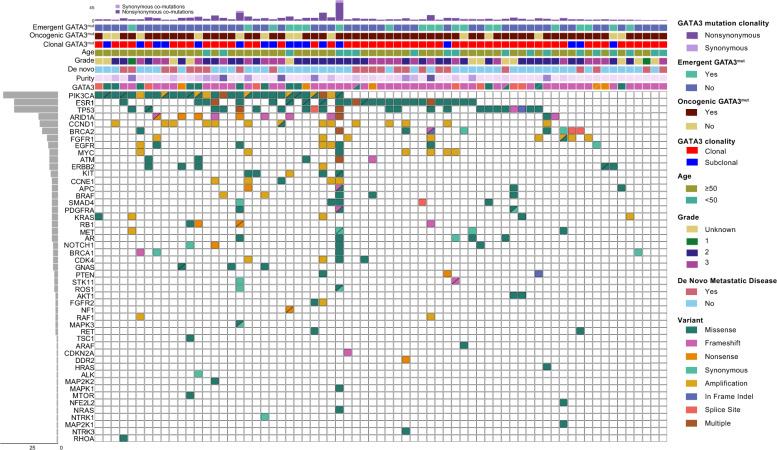
CoMut plot of *GATA3* and other gene mutations in our study population

In 3 of the 69 patients with *GATA3*.^*mut*^ HR +/HER2− MBC, *GATA3* was the only detectable ctDNA alteration, while remaining patients had concomitant co-mutations with a median number of 4 variants (range 1–44) (Fig. 2). The most frequent co-mutations were in *PIK3CA* (*n* = 30), *ESR1* (*n* = 26), and *TP53* (*n* = 23), and the most frequent amplification was in *CCND1* (*n* = 14). There were no statistically significant associations (positive or negative) between *GATA3* and *TP53* (Fisher exact *p* = 0.30)*,* and *GATA3* and *PIK3CA* (*p* = 0.52). These findings are distinct from the co-mutational landscapes based on tissue analyses of primary breast cancers, where *GATA3* mutations appeared to be mutually exclusive from both *TP53* and *PIK3CA* mutations [14, 25, 33].

In the ctDNA-detected *GATA3*^*mut*^ population, *TP53* co-mutations (*n* = 23) were found with a median VAF of 1.0% (range 0.03–30.5%). Of note, plasma-based cell-free DNA assays cannot definitively distinguish tumor-derived somatic mutations from clonal hematopoiesis (CH), in which *TP53* is a commonly mutated gene [49]. 55 of the 69 patients (80%) had predicted clonal *GATA3* mutations (based on above criteria), and 12/23 (52%) *TP53* co-mutations appeared to be clonal. Among the clonal *TP53* variants, 8 (67%) had a VAF > 1%. 5/12 patients had both *TP53* and *GATA3* as suspected clonal variants, only 2 of whom also had a VAF > 1% (Supplemental Fig. 2).

Within the *GATA3*^*mut*^ population, *GATA3* was the mutation with the highest VAF in 23 patients (34% of patients). Among patients where *GATA3* was not the highest VAF, the other highest mutations included *PIK3CA* (*n* = 19), *ESR1* (*n* = 7), *TP53* (*n* = 8), *BRCA1* (*n* = 2), *BRCA2* (*n* = 2), and *SMAD4* (*n* = 2).

Interestingly, there were 3 patients with germline mutations in *BRCA1/2*. As mentioned above, the Guardant 360 assay does not report germline mutations, but suspicion is raised when allele fraction is above 40% (and much higher than other mutations). By this criterion, 3 of the 7 (43%) patients with non-synonymous *BRCA1/2* mutations were noted to have findings suspicious for germline variants, which was later confirmed on retrieval of documented genetic testing. This appears to be a new finding compared to an earlier study of *GATA3* mutations in familial breast cancer, where *GATA3* mutations were found in 22% (7/32) of patients without BRCA1/1 mutations and not identified in patients with germline *BRCA1* or *BRCA2* mutations (*n* = 0/23) [50].

Among the 69 patients with *GATA3*^*mut*^ HR +/HER2− MBC, there were 20 patients with serial sampling data. Among these, in 5 patients, *GATA3* mutation(s) had not been identified on initial ctDNA sampling but rather were identified on subsequent draws. New detection on serial sampling may be due to one of several factors: emergence of a new variant, or else the variant existing in the first sample but below the limit of detection. While the numbers are relatively small, many of the *GATA3* frameshift mutations appeared to increase in serial sampling in synchrony with known driver alterations such as *PIK3CA* or *ESR1,* as well as *ARID1A* and *ATM,* though disparate growth patterns were also observed in 3 patients. (Supplemental Fig. 3).

**Fig. 3:**
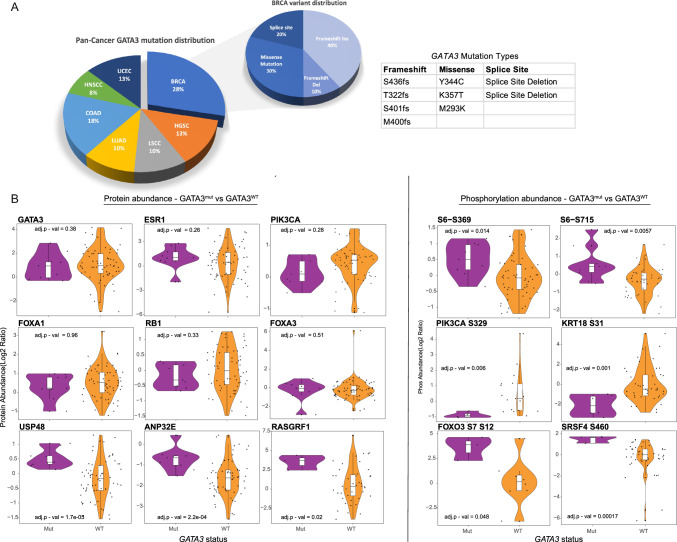
GATA3 proteomic profiling using CPTAC database—**A** *GATA3* mutation and variant distribution; **B** Proteomic and phosphoproteomic expression changes between *GATA3*^*WT*^ vs *GATA3*^mut^ samples

### *GATA3* mutations and proteomic correlates

To understand the functional implications of *GATA3* mutations, the National Cancer Institute's tissue-based Clinical Proteomic Tumor Analysis Consortium (CPTAC) database was interrogated for HR +/HER2− breast cancers harboring *GATA3* mutations, and the expression and phosphorylation of *GATA3* and associated proteins were analyzed. Among 121 patients with breast cancer, 10 patients had a single *GATA3* mutation. We identified 6 luminal A, 3 luminal B, and 1 HER2-enriched cases. The latter was excluded given the focus of this analysis on HR +/HER2− breast cancer. Among the remaining 9 *GATA3-*mutated, there were 4 frameshift, 3 missense, and 2 splice site variants. We did not detect differences in *GATA3* protein abundance when comparing *GATA3*-mutated to HR +/HER2− unmutated samples (Fig. 3). The frameshift mutations were located in the C-terminal domain and have not been demonstrated to impact protein levels of function. [51] Similarly, there was no difference in protein abundance of breast cancer-associated genes, including *ESR1, PIK3CA, FOXA1, FOXO3*, or *RB1* (0.2 < FDR < 0.9). Notably, *GATA3* mutations were associated with a significant increase in abundance of the deubiquitinating enzyme USP48 (LogFC = 0.76, FDR = 1.7 × 10^–5^), which stabilizes MDM2, and thus enhances p53 ubiquitination and degradation [52]. All above samples with *GATA3* mutations had wildtype *TP53.*

Global differential expression analysis of phosphorylation sites revealed a significant increase of RPS6 KA3 phosphorylation levels on sites S369 and S715 (FDR = 0.014 & 0.006 respectively), which both reside on the highly conserved catalytic domain of the S6 kinase, a component of the RAS/ERK signaling pathway. Similarly, we saw increased phosphorylation on FOXO3 (Sites S7 and S12, FDR = 0.048), both suggesting increased downstream signaling, potentially via ERK. KRT18 showed significantly reduced phosphorylation at multiple sites (such as S31, FDR = 0.001); this protein is linked with the epithelial-mesenchymal transition and was explored given the association of *GATA3* mutations and metastasis. In addition, the histone chaperone ANP32E, which has been shown to be inversely correlated with tumor progression and relaxation of chromatin at FOXA1 binding sites, was found to be higher in *GATA3*^mut^ cancers, which is in keeping with published literature (LogFC = 1.09, FDR = 2.2 × 10^–4^) [53]. Activation of these pathways may lead to estrogen receptor signaling independence.

### *GATA3* mutations and clinical outcomes

Finally, we evaluated the association of ctDNA-detected *GATA3* mutations with clinical outcomes in the HR +/HER2− MBC setting, stratified by type of therapy. Among patients who received endocrine monotherapy (ET; *GATA3*^*WT*^, *n* = 74, *GATA3*^*mut*^*,* *n* = 6), *GATA3*^*mut*^ were associated with worse progression-free survival (PFS; *p* = 0.061) and worse overall survival (OS; *p* = 0.004); sample sizes were not powered to detect statistically significant differences. There was no statistically significant difference in PFS or OS between GATA3^mut^ (*n* = 25) and GATA3^*WT*^ (*n* = 188) subgroups that received chemotherapy and those that received ET + CDK4/6 inhibitor treatment.

**Fig. 4:**
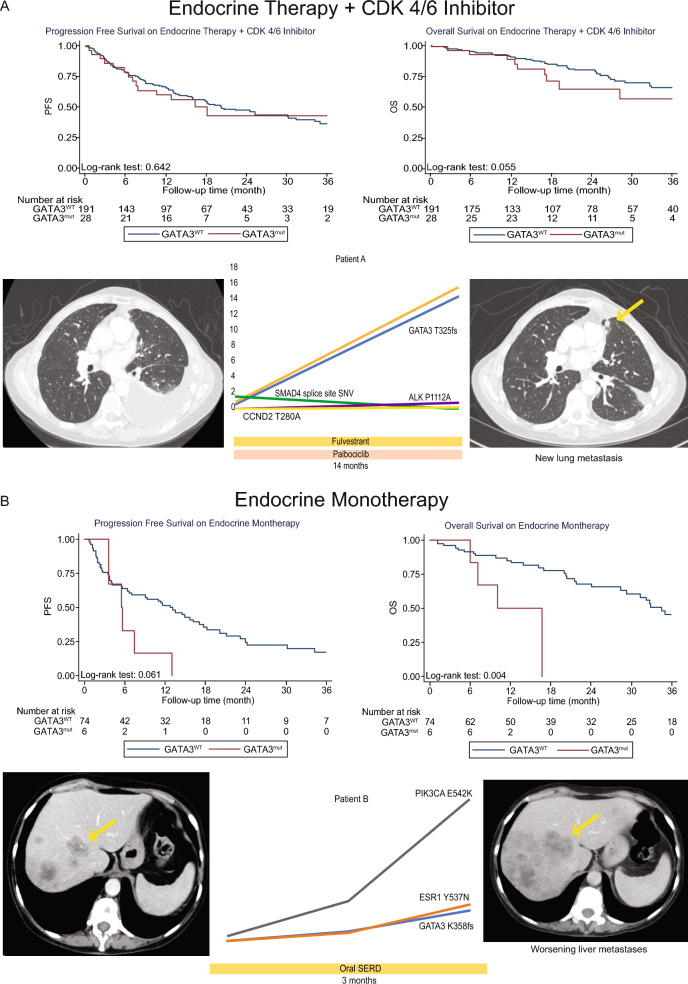
Survival analyses of *GATA3 mut* patients treated with ET + CDK4/6 and ET alone

In two contrasting index patient cases, we describe this clinical story at the individual level along with ctDNA clonal pattern. One patient with known lung metastases treated with palbociclib in combination with fulvestrant, remained on this therapy for 14 months, during which *GATA3* T329fs and *ESR1* D538G emerged, likely both in a resistant dominant clone (Fig. 4a). The disease remained stable for over a year of therapy, after which the patient developed a new lung metastasis. In contrast, in Fig. 4d, a patient with known liver metastases was treated with a novel oral selective estrogen receptor degrader (SERD) monotherapy. The liver metastases increased in size and number after only 3 months. During this time, there was a steady increase in the allele fractions in 3 mutations: *PIK3CA* E542 K, *ESR1* Y537 N, and *GATA3* K358f in a manner suggesting the *PIK3CA* mutation was clonal, and the *ESR1* and *GATA3* variants were subclonal. No amplifications in *PIK3CA* were detected.

## Discussion

Numerous sequencing analyses have shown that *GATA3* gene mutations are common in breast cancer [33–36, 54–56]. Despite this fact, the GATA3 protein has not yet been found to be therapeutically targetable, directly or indirectly. As new approaches to target *GATA3*^*mut*^ breast cancer are underway, it is important to further broaden our understanding of the mutational landscape of such disease. To date, the majority of large-scale genomic studies have been performed via tissue-based analyses in primary breast cancer. With this study, we add a new dimension to the *GATA3*^*mut*^ landscape by analyzing ctDNA in MBC and demonstrate that *GATA3* mutations are detectable, as well as co-occurring potential modifiers, and cancers harboring these mutations have distinct ctDNA-based genomic characteristics compared to those identified in primary breast cancer tissue. Specifically, ctDNA analysis in the advanced breast cancer setting showed that *GATA3* mutations are not mutually exclusive from *TP53* or *PIK3CA* mutations. This discrepancy may be explained by a number of different factors: biologic differences in advanced disease, disparate clonal evolution at different metastatic sites captured in a single blood draw, differences in variant detectability via blood compared to tissue, and/or contributions from clonal hematopoiesis (unrelated to tumor evolution) in this study population, where the median age was > 55.

Our tissue-based proteomic analysis highlighted important potential contributors to estrogen pathway independence, as well as a mechanistic explanation for sensitivity to MDM2 inhibition. While GATA3, ESR1, PIK3CA, FOXA1, FOXO3, and RB1 protein abundances were not significantly reduced in *GATA3*^mut^ breast cancers, the expression of genes associated with MDM2 function and phosphorylation of genes associated with ERK signaling and aggressive phenotypes were impacted. The finding of increased abundance of USP48 protein, which was explored given the potential mechanistic link between MDM2 inhibition efficacy in *GATA3*^mut^ breast cancer, supported the hypothesis that *GATA3* mutations were leading to changes in the MDM2 signaling pathway, which in turn lead to the therapeutic vulnerability of MDM2 inhibition. [31] Moreover, differential expression analysis of phosphorylation sites revealed a potential association of increased downstream signaling via ERK, which could also be leveraged for potential therapeutic inhibition.

The strong pre-clinical data around MDM2 inhibition’s potential synthetic lethality role in ER +, *GATA3*^mut^ ER + breast cancer led to the design of a phase II clinical trial using the MDM2 inhibitor milademetan plus fulvestrant in patients with ER +, HER2−, *GATA3*^mut^ advanced breast cancer (NCT05932667), where the *GATA3* mutation would be identified via sequencing the tumor and/or ctDNA. Patients must have wildtype *TP53*, and our findings suggest screening via ctDNA may find a smaller population of candidate patients, given the lack of mutual exclusivity of *GATA3* and *TP53* ctDNA mutations in this population with advanced disease.

Meanwhile, 3 patients with ctDNA-detected *GATA3* mutations in our study also had germline *BRCA1/2* mutations, which has not been previously appreciated. [50] These findings highlight likely functional heterogeneity among mutations in different regions of the *GATA3* gene. Furthermore, pathogenic germline *BRCA1/2* variants qualify patients for PARP inhibitor therapy, which offers alternative targeted therapy options to patients who may otherwise be considered for a *GATA3*^mut^ targeted clinical trial [57, 58].

Our study also highlights the data we may gain from serial sampling. *GATA3* mutations in 5 patients were not initially identified on blood sampling, and only through serial sampling over time were these found. This emergence of *GATA3* mutations may suggest their role in modifying therapeutic response. The importance of serial testing at multiple timepoints throughout treatment is increasing in MBC, where we may identify emergence of dynamic key genomic changes that can drive both resistance and sensitivity to therapeutic agents, such as *ESR1, PIK3CA, PTEN, AKT1,* and others; *GATA3* mutations were also shown in this dataset to co-occur with alterations in many of these genes at high frequency. *GATA3* mutations did not exhibit consistent mutational dynamics over time, which may speak to multiple factors, including treatment effect and/or resistance within certain (sub)clones, but also a likely non-uniform functional nature of different *GATA3* mutations, as noted above. Our findings allude to differential clonal dynamics in which *GATA3* variants do not always follow mutational patterns of known driver variants such as *PIK3CA*, which is typically clonal, nor *ESR1*, which is typically subclonal (Supplemental Fig. 3). This data require further exploration in larger and prospective ctDNA-based studies.

Survival analyses were consistent with published results from a subgroup analysis in the MONALEESA-7 trial assessing ribociclib + ET in premenopausal patients, where *GATA3* mutations were associated with poorer survival in patients receiving ET monotherapy but not in patients receiving ribociclib + ET [59]. Our findings are also interesting considering early data suggesting *GATA3* mutations may predict sensitivity to monotherapy with the CDK4/6 inhibitor abemaciclib [60]. Importantly, prognostic and predictive findings are not necessarily linked, thus the efficacy of targeted therapy in this population remains to be explored in clinical trials.

While ctDNA offers the ease of serial sampling and evaluation of tumor DNA content across metastatic sites, there are also key limitations of this tool. As described above, clonal hematopoiesis is a major potential confounder to consider in using this toolset. Furthermore, low ctDNA fractions would limit the sensitivity of the NGS assays to detect variants of interest, as well as physical barriers to collection, such as the blood–brain barrier. This study is also retrospective and does not analyze matched tissue samples collected at the time of ctDNA analysis. This is an important limitation of this study. Further confounders may include the effects of prior lines of therapy, variation in the timing and clinical contexts of sample collection, and the impacts of ongoing therapy at the time of collection.

## Conclusion

Accruing knowledge on the nature of *GATA3* gene mutations in breast cancer offers hope for a new arsenal of targeted therapies. To date, this transcription factor has remained an area of great interest but has not yet proven therapeutically actionable. As tumor and plasma genomic profiling are becoming available to a wider population of patients via universal genomic testing initiatives, we may further define the genomic landscape of *GATA3* mutations in breast cancer. ctDNA can be used to evaluate breast cancer genomics at different time points, which we may allow real time assessment of *GATA3*^mut^ breast cancer biology and response to therapy while searching for targetable variants in this promising population.

## Supplementary Information

Below is the link to the electronic supplementary material.

Supplementary file1 (DOCX 537 KB)

## Data Availability

Data is provided within the manuscript or supplementary information files.

## References

[1] Siegel RL, Miller KD, Fuchs HE, Jemal A (2022) Cancer statistics, 2022. CA Cancer J Clin 72:7–33 35020204 10.3322/caac.21708

[2] André F, Ciruelos E, Rubovszky G et al (2019) Alpelisib for PIK3CA-mutated, hormone receptor-positive advanced breast cancer. N Engl J Med 380:1929–1940 31091374 10.1056/NEJMoa1813904

[3] Bidard F-C, Kaklamani VG, Neven P et al (2022) Elacestrant (oral selective estrogen receptor degrader) versus standard endocrine therapy for estrogen receptor-positive, human epidermal growth factor receptor 2-negative advanced breast cancer: results from the randomized phase III EMERALD trial. J Clin Oncol 40:3246–3256 35584336 10.1200/JCO.22.00338 PMC9553388

[4] Turner NC, Oliveira M, Howell SJ et al (2023) Capivasertib in hormone receptor-positive advanced breast cancer. N Engl J Med 388:2058–2070 37256976 10.1056/NEJMoa2214131 PMC11335038

[5] Velimirovic M, Gerratana L, Davis AA et al (2021) Landscape of GATA3 mutations identified from circulating tumor DNA clinical testing and their impact on disease outcomes in estrogen receptor-positive (ER+) metastatic breast cancers treated with endocrine therapies. J Clin Oncology 39:1065–1065

[6] Theodorou V, Stark R, Menon S, Carroll JS (2013) GATA3 acts upstream of FOXA1 in mediating ESR1 binding by shaping enhancer accessibility. Genome Res 23:12–22 23172872 10.1101/gr.139469.112 PMC3530671

[7] Kong SL, Li G, Loh SL et al (2011) Cellular reprogramming by the conjoint action of ERα, FOXA1, and GATA3 to a ligand-inducible growth state. Mol Syst Biol 7:526 21878914 10.1038/msb.2011.59 PMC3202798

[8] Kouros-Mehr H, Bechis SK, Slorach EM et al (2008) GATA-3 links tumor differentiation and dissemination in a luminal breast cancer model. Cancer Cell 13:141–152 18242514 10.1016/j.ccr.2008.01.011 PMC2262951

[9] Kouros-Mehr H, Slorach EM, Sternlicht MD, Werb Z (2006) GATA-3 maintains the differentiation of the luminal cell fate in the mammary gland. Cell 127:1041–1055 17129787 10.1016/j.cell.2006.09.048 PMC2646406

[10] Dydensborg AB, Rose AAN, Wilson BJ et al (2009) GATA3 inhibits breast cancer growth and pulmonary breast cancer metastasis. Oncogene 28:2634–2642 19483726 10.1038/onc.2009.126

[11] Asselin-Labat M-L, Sutherland KD, Barker H et al (2007) Gata-3 is an essential regulator of mammary-gland morphogenesis and luminal-cell differentiation. Nat Cell Biol 9:201–209 17187062 10.1038/ncb1530

[12] Ciocca V, Daskalakis C, Ciocca RM et al (2009) The significance of GATA3 expression in breast cancer: a 10-year follow-up study. Hum Pathol 40:489–495 19084267 10.1016/j.humpath.2008.09.010

[13] Du F, Yuan P, Wang T et al (2015) The significance and therapeutic potential of GATA3 expression and mutation in breast cancer: a systematic review. Med Res Rev 35:1300–1315 26313026 10.1002/med.21362

[14] Ping Z, Xia Y, Shen T et al (2016) A microscopic landscape of the invasive breast cancer genome. Sci Rep 6:27545 27283966 10.1038/srep27545 PMC4901326

[15] Shan L, Li X, Liu L et al (2014) GATA3 cooperates with PARP1 to regulate CCND1 transcription through modulating histone H1 incorporation. Oncogene 33:3205–3216 23851505 10.1038/onc.2013.270

[16] Cohen H, Ben-Hamo R, Gidoni M et al (2014) Shift in GATA3 functions, and GATA3 mutations, control progression and clinical presentation in breast cancer. Breast Cancer Res 16:464 25410484 10.1186/s13058-014-0464-0 PMC4303202

[17] Giuliano M, Schettini F, Rognoni C et al (2019) Endocrine treatment versus chemotherapy in postmenopausal women with hormone receptor-positive, HER2-negative, metastatic breast cancer: a systematic review and network meta-analysis. Lancet Oncol 20:1360–1369 31494037 10.1016/S1470-2045(19)30420-6

[18] Bertucci F, Ng CKY, Patsouris A et al (2019) Genomic characterization of metastatic breast cancers. Nature 569:560–564 31118521 10.1038/s41586-019-1056-z

[19] Mehra R, Varambally S, Ding L et al (2005) Identification of GATA3 as a breast cancer prognostic marker by global gene expression meta-analysis. Cancer Res 65:11259–11264 16357129 10.1158/0008-5472.CAN-05-2495

[20] Gulbahce HE, Sweeney C, Surowiecka M et al (2013) Significance of GATA-3 expression in outcomes of patients with breast cancer who received systemic chemotherapy and/or hormonal therapy and clinicopathologic features of GATA-3-positive tumors. Hum Pathol 44:2427–2431 23998430 10.1016/j.humpath.2013.05.022

[21] Larsen V, Barlow WE, Yang JJ et al (2019) Germline genetic variants in GATA3 and breast cancer treatment outcomes in SWOG S8897 trial and the pathways study. Clin Breast Cancer 19:225-235.e2 30928413 10.1016/j.clbc.2019.02.010 PMC6667288

[22] Takaku M, Grimm SA, Wade PA (2015) GATA3 in breast cancer: tumor suppressor or oncogene? Gene Expr 16:163–168 26637396 10.3727/105221615X14399878166113 PMC4758516

[23] Voduc D, Cheang M, Nielsen T (2008) GATA-3 expression in breast cancer has a strong association with estrogen receptor but lacks independent prognostic value. Cancer Epidemiol Biomarkers Prev 17:365–373 18268121 10.1158/1055-9965.EPI-06-1090

[24] Gruvberger S, Ringnér M, Chen Y et al (2001) Estrogen receptor status in breast cancer is associated with remarkably distinct gene expression patterns. Cancer Res 61:5979–5984 11507038

[25] Jiang Y-Z, Yu K-D, Zuo W-J et al (2014) GATA3 mutations define a unique subtype of luminal-like breast cancer with improved survival. Cancer 120:1329–1337 24477928 10.1002/cncr.28566

[26] Hoch RV, Thompson DA, Baker RJ, Weigel RJ (1999) GATA-3 is expressed in association with estrogen receptor in breast cancer. Int J Cancer 84:122–128 10096242 10.1002/(sici)1097-0215(19990420)84:2<122::aid-ijc5>3.0.co;2-s

[27] Perou CM, Sørlie T, Eisen MB et al (2000) Molecular portraits of human breast tumours. Nature 406:747–752 10963602 10.1038/35021093

[28] Sorlie T, Tibshirani R, Parker J et al (2003) Repeated observation of breast tumor subtypes in independent gene expression data sets. Proc Natl Acad Sci USA 100:8418–8423 12829800 10.1073/pnas.0932692100 PMC166244

[29] Sotiriou C, Pusztai L (2009) Gene-expression signatures in breast cancer. N Engl J Med 360:790–800 19228622 10.1056/NEJMra0801289

[30] Jenssen T-K, Kuo WP, Stokke T, Hovig E (2002) Associations between gene expressions in breast cancer and patient survival. Hum Genet 111:411–420 12384785 10.1007/s00439-002-0804-5

[31] Bianco G, Coto-Llerena M, Gallon J et al (2022) GATA3 and MDM2 are synthetic lethal in estrogen receptor-positive breast cancers. Communications Biology 5:1–15 35440675 10.1038/s42003-022-03296-x PMC9018745

[32] Ciriello G, Gatza ML, Beck AH et al (2015) Comprehensive molecular portraits of invasive lobular breast cancer. Cell 163:506–519 26451490 10.1016/j.cell.2015.09.033 PMC4603750

[33] Ellis MJ, Ding L, Shen D et al (2012) Whole-genome analysis informs breast cancer response to aromatase inhibition. Nature 486:353–360 22722193 10.1038/nature11143 PMC3383766

[34] Nik-Zainal S, Davies H, Staaf J et al (2016) Landscape of somatic mutations in 560 breast cancer whole-genome sequences. Nature 534:47–54 27135926 10.1038/nature17676 PMC4910866

[35] Stephens PJ, Tarpey PS, Davies H et al (2012) The landscape of cancer genes and mutational processes in breast cancer. Nature 486:400–404 22722201 10.1038/nature11017 PMC3428862

[36] Pereira B, Chin S-F, Rueda OM et al (2016) The somatic mutation profiles of 2,433 breast cancers refines their genomic and transcriptomic landscapes. Nat Commun 7:11479 27161491 10.1038/ncomms11479 PMC4866047

[37] Razavi P, Chang MT, Xu G et al (2018) The genomic landscape of endocrine-resistant advanced breast cancers. Cancer Cell 34:427-438.e6 30205045 10.1016/j.ccell.2018.08.008 PMC6327853

[38] Afzaljavan F, Sadr AS, Savas S, Pasdar A (2021) GATA3 somatic mutations are associated with clinicopathological features and expression profile in TCGA breast cancer patients. Sci Rep 11:1679 33462316 10.1038/s41598-020-80680-9 PMC7814117

[39] Burstein HJ, DeMichele A, Somerfield MR et al (2023) Testing for ESR1 mutations to guide therapy for hormone receptor-positive, human epidermal growth factor receptor 2-negative metastatic breast cancer: ASCO guideline rapid recommendation update. J Clin Oncol 41:3423 37196213 10.1200/JCO.23.00638

[40] Chakravarty D, Johnson A, Sklar J et al (2022) Somatic genomic testing in patients with metastatic or advanced cancer: ASCO provisional clinical opinion. J Clin Oncol 40:1231 35175857 10.1200/JCO.21.02767

[41] Henry NL, Somerfield MR, Dayao Z et al (2022) Biomarkers for systemic therapy in metastatic breast cancer: ASCO GUIDELINE UPdate. J Clin Oncol 40:3205 35759724 10.1200/JCO.22.01063

[42] Odegaard JI, Vincent JJ, Mortimer S et al (2018) Validation of a plasma-based comprehensive cancer genotyping assay utilizing orthogonal tissue- and plasma-based methodologies. Clin Cancer Res 24:3539–3549 29691297 10.1158/1078-0432.CCR-17-3831

[43] Chakravarty D, Gao J, Phillips SM et al (2017) OncoKB: a precision oncology knowledge base. JCO Precis Oncol. 10.1200/PO.17.00011. 28890946 10.1200/PO.17.00011 PMC5586540

[44] Geffen Y, Anand S, Akiyama Y et al (2023) Pan-cancer analysis of post-translational modifications reveals shared patterns of protein regulation. Cell 186:3945-3967.e26 37582358 10.1016/j.cell.2023.07.013 PMC10680287

[45] Ali A, Christie PT, Grigorieva IV et al (2007) Functional characterization of GATA3 mutations causing the hypoparathyroidism-deafness-renal (HDR) dysplasia syndrome: insight into mechanisms of DNA binding by the GATA3 transcription factor. Hum Mol Genet 16:265–275 17210674 10.1093/hmg/ddl454

[46] Mair B, Konopka T, Kerzendorfer C et al (2016) Gain- and loss-of-function mutations in the breast cancer gene GATA3 result in differential drug sensitivity. PLoS Genet 12:e1006279 27588951 10.1371/journal.pgen.1006279 PMC5010247

[47] Barakat AJ, Raygada M, Rennert OM (2018) Barakat syndrome revisited. Am J Med Genet A 176:1341–1348 29663634 10.1002/ajmg.a.38693

[48] Van Esch H, Groenen P, Nesbit MA et al (2000) GATA3 haplo-insufficiency causes human HDR syndrome. Nature 406:419–422 10935639 10.1038/35019088

[49] Jaiswal S, Fontanillas P, Flannick J et al (2014) Age-related clonal hematopoiesis associated with adverse outcomes. N Engl J Med 371:2488–2498 25426837 10.1056/NEJMoa1408617 PMC4306669

[50] Arnold JM, Choong DYH, Thompson ER et al (2010) Frequent somatic mutations of GATA3 in non-BRCA1/BRCA2 familial breast tumors, but not in BRCA1-, BRCA2- or sporadic breast tumors. Breast Cancer Res Treat 119:491–496 19189213 10.1007/s10549-008-0269-x

[51] Gustin JP, Miller J, Farag M et al (2017) GATA3 frameshift mutation promotes tumor growth in human luminal breast cancer cells and induces transcriptional changes seen in primary GATA3 mutant breast cancers. Oncotarget 8:103415–103427 29262572 10.18632/oncotarget.21910 PMC5732738

[52] Cetkovská K, Šustová H, Uldrijan S (2017) Ubiquitin-specific peptidase 48 regulates Mdm2 protein levels independent of its deubiquitinase activity. Sci Rep 7:43180 28233861 10.1038/srep43180 PMC5324091

[53] Ruff GL, Murphy KE, Smith ZR et al (2021) Subtype-independent ANP32E reduction during breast cancer progression in accordance with chromatin relaxation. BMC Cancer 21:1342 34922480 10.1186/s12885-021-09077-9 PMC8684129

[54] Banerji S, Cibulskis K, Rangel-Escareno C et al (2012) Sequence analysis of mutations and translocations across breast cancer subtypes. Nature 486:405–409 22722202 10.1038/nature11154 PMC4148686

[55] Cancer Genome Atlas Network (2012) Comprehensive molecular portraits of human breast tumours. Nature 490:61–70 23000897 10.1038/nature11412 PMC3465532

[56] Usary J, Llaca V, Karaca G et al (2004) Mutation of GATA3 in human breast tumors. Oncogene 23:7669–7678 15361840 10.1038/sj.onc.1207966

[57] Robson M, Im S-A, Senkus E, Xu B, Domchek SM, Masuda N, Delaloge S, Li W, Tung N, Armstrong A, Wu W, Goessl C, Runswick S, Conte P (2017) Olaparib for metastatic breast cancer in patients with a germline BRCA mutation. N Engl J Med 377(6):523–533 28578601 10.1056/NEJMoa1706450

[58] Litton JK, Rugo HS, Ettl J, Hurvitz SA, Gonçalves A, Lee K-H, Fehrenbacher L, Yerushalmi R, Mina LA, Martin M, Roché H, Im Y-H, Quek RGW, Markova D, Tudor IC, Hannah AL, Eiermann W, Blum JL (2018) Talazoparib in patients with advanced breast cancer and a germline BRCA mutation. N Engl J Med 379(8):753–763 30110579 10.1056/NEJMoa1802905 PMC10600918

[59] Bardia A, Su F, Solovieff N et al (2021) Genomic profiling of premenopausal HR+ and HER2− metastatic breast cancer by circulating tumor DNA and association of genetic alterations with therapeutic response to endocrine therapy and ribociclib. JCO Precis Oncol. 10.1200/PO.20.00445 34504990 10.1200/PO.20.00445 PMC8423397

[60] Hamilton EP, Cortes J, Jerusalem G et al (2020) Abstract 785: Genomic markers of response to monotherapy abemaciclib in the nextMONARCH 1 study. Cancer Res 80:785–785

