# Supplementary material for: Genomic and proteomic profiling of GATA3 mutant metastatic hormone receptor-positive breast cancer and impact on clinical outcomes
Source: Breast Cancer Res Treat. 2025 May 29;212(3):437–47. doi: 10.1007/s10549-025-07710-w (PMC12209021; doi:10.1007/s10549-025-07710-w)

**Supplemental Table 1:** Patient characteristics in patients with likely oncogenic & oncogenic *GATA3* mutations compared to patients *GATA3* wildtype disease.

**Supplemental Figure 1.** Distribution of gene mutations in the Catalog Of Somatic Mutations In Cancer (COSMIC) database. Database can be accessed at: https://cancer.sanger.ac.uk/cosmic.
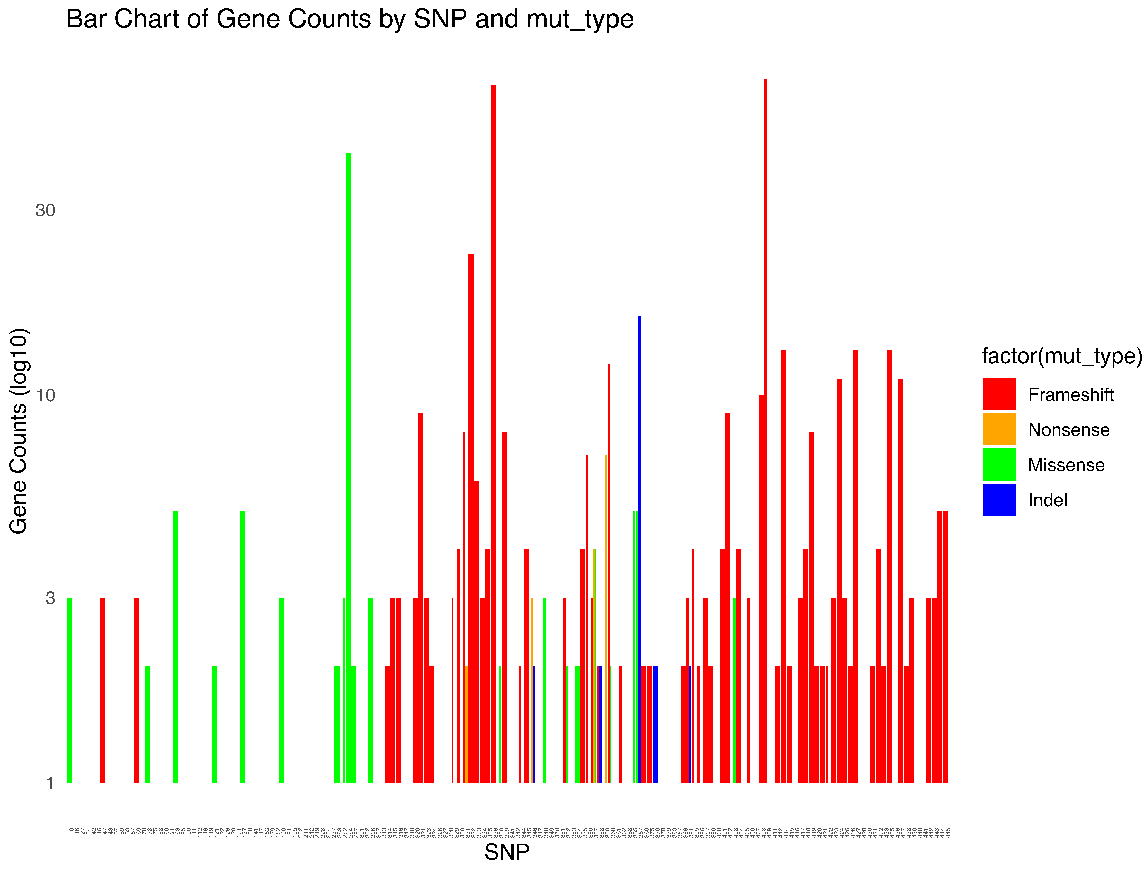
**Supplemental Figure 2.** Variant allele fraction of co-occurring *GATA3* and *TP53* variants in individual patient samples.


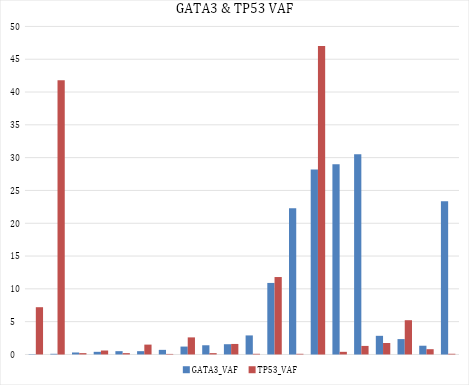


**Supplemental Figure 3.** Examples of temporal clonal dynamics of 9 patients with GATA detected via serial ctDNA sampling.


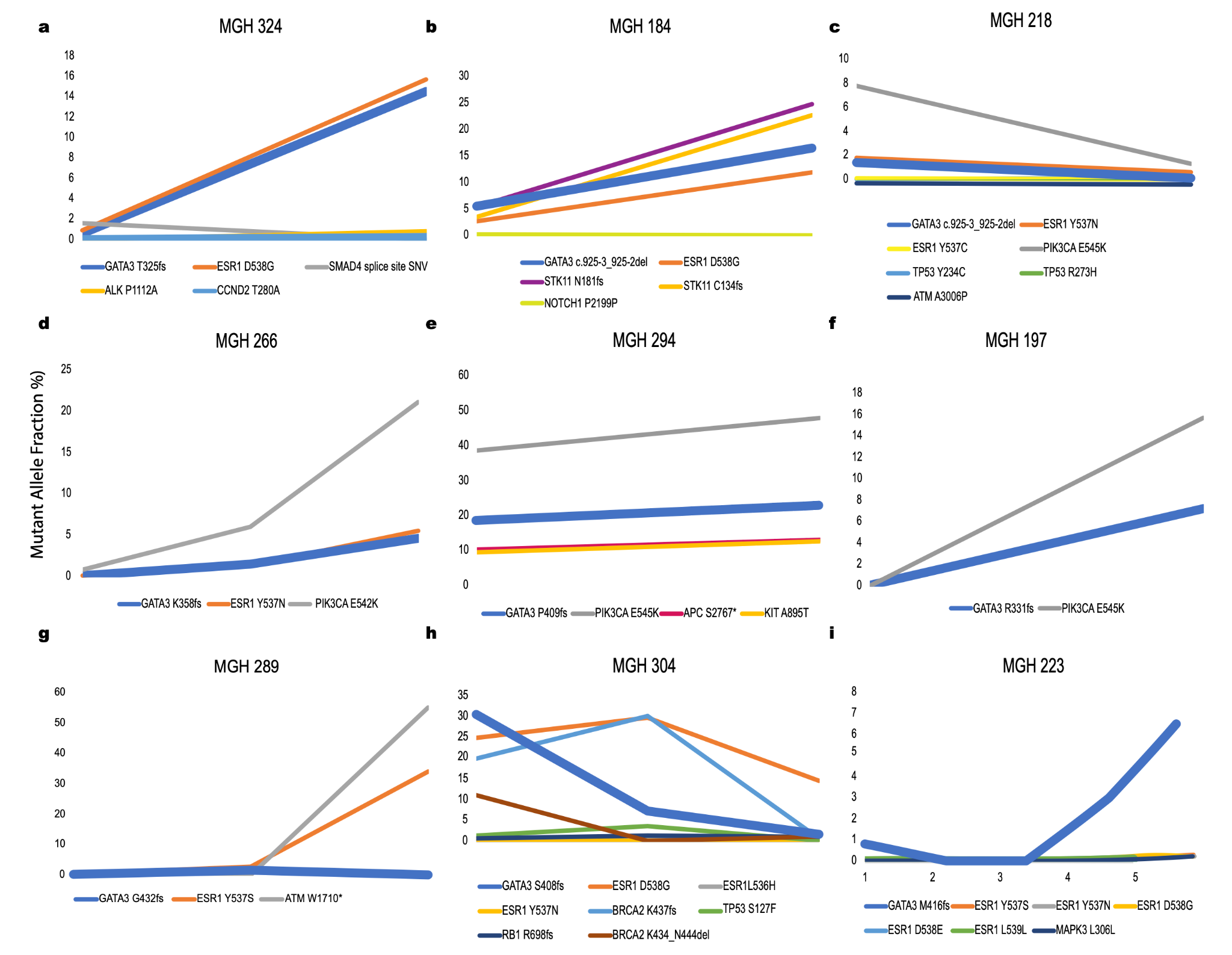

Supplement: Supplementary file 1 — Supplementary file1 (DOCX 537 KB) [file 10549_2025_7710_MOESM1_ESM.docx]
